# Translation, cultural adaptation and validation of the Somatic Symptom Scale-8 (SSS-8) for the Brazilian Portuguese language

**DOI:** 10.1186/s12875-022-01836-2

**Published:** 2022-09-05

**Authors:** Camila Fernandes Pollo, Silmara Meneguin, Hélio Amante Miot, César de Oliveira

**Affiliations:** 1 Nursing Department, Faculdade de Medicina de Botucatu, Universidade Estadual Paulista (FMB-Unesp), Emilio Garcia, 464, Jardim Bom Pastor, Botucatu, SP CEP 18603-440 Brazil; 2 Department of Infectology, Dermatology, Radiotherapy and Imaging Diagnosis, Faculdade de Medicina de Botucatu, Universidade Estadual Paulista (FMB-Unesp), Botucatu, SP Brazil; 3 Department of Epidemiology & Public Health, University College London, London, UK

**Keywords:** Questionnaires, Quality of life, Dermatology, Somatic symptoms, Psychometry

## Abstract

**Background:**

Assessment tools are commonly used in different fields of health to assist in the diagnosis, the evaluation of the response to treatment, the measurement of quality of life and the establishment of the prognosis.

**Objectives:**

Translate, culturally adapt and perform the psychometric validation of the Somatic Symptom Scale (SSS-8) for use in the Brazilian population.

**Methods:**

Cross-cultural adaptation followed a combination of guidelines and for psychometric evaluation a sample of 300 patients was recruited. All recommended measurement properties by the Consensus-based Standards for the selection of health status Measurement Instruments were evaluated, including analysis by an expert committee and analysis by the target public. The Skindex-16 was used for the evaluation of convergent validity and Cronbach’s alpha was used for the determination of the internal consistency of the translated version of the SSS-8.

**Results:**

The final version received approval from five experts and the agreement index was 100% for all items. During the pretest, the scale was administered to 300 patients with psoriasis and none of the items needed to be altered. A moderate correlation was found between the Skindex-16 and SSS-8-BRA. In the analysis of internal consistency, Cronbach’s alpha for the SSS-8-BRA was 0.81.

**Conclusion:**

The SSS-8 is a valid and reliable tool for the assessment of somatic symptoms in the Brazilian population.

**Supplementary Information:**

The online version contains supplementary material available at 10.1186/s12875-022-01836-2.

## Key message

The present study involved the translation, cultural adaption and determination of the psychometric properties of the SSS-8-BRA. This scale is potentially useful in numerous medical situations, as the scores can serve as quantitative markers of the burden of somatic symptoms in patients with chronic conditions.

## Background

Assessment tools are commonly used in different fields of health to assist in the diagnosis, the evaluation of the response to treatment, the measurement of quality of life and the establishment of the prognosis [1]. However, the choice of the ideal assessment tool requires considering components such as clarity, simplicity, comprehension, application and administration time [2]. When an instrument is designed, its measurement properties need to be tested and validated in a group of patients before being used in population groups [3]. Moreover, the psychometric performance of health measures varies between populations, which can limit their usefulness in some contexts [4].

In this process, translation and validation are needed when the aim is to use an assessment tool originally created by researchers in a different country with different characteristics. However, the validity and reliability of the new version are dependent on following well-defined scientific procedures [5]. This process has been used in several fields of knowledge and has benefits over the creation of a new instrument, such as reductions in cost and time and the possibility of comparing different contexts.

The original version of the Somatic Symptom Scale (SSS-8) was developed in Germany in 2014 as a brief outcome measure of the burden of somatic symptoms reported by patients in the general population and was subsequently translated and validated for the Greek, Japanese and Iranian populations [6–9].

For the present study, the sample was composed entirely of patients with psoriasis, which is a chronic immune-mediated inflammatory disease that affects approximately 2% of the world population [10, 11]. Clinically, the classic form of the disease is characterized by erythematous plaques covered by greyish-white scales, most commonly on the elbows and knees. However, psoriasis is not only a dermatological disease; it is also a systemic condition [12].

The occurrence of an important comorbidity, such as psoriasis, exerts a negative impact on quality of life, as it is associated with functional decline and a scenario of intense pain, depending on the way the disease manifests itself [13]. Symptoms constitute one of the major complaints of patients with chronic diseases, but the intensity of the complaint does not always correspond to clinical findings identified through a medical evaluation, underscoring the need to consider psychosocial factors as criteria for the analysis and quantification of the impact of a painful condition [14].

The impact of somatic symptoms on the quality of life of individuals with psoriasis is an important component to evaluate for a better understanding of the behavior of the disease. Moreover, greater knowledge on pain complaints could enable the establishment of more adequate strategies to meet the needs of patients. Therefore, the aim of the present study was to translate, culturally adapt and perform the psychometric validation of the Somatic Symptom Scale (SSS-8) for the Brazilian population.

## Methods

A methodological study was conducted involving the translation, adaptation and validation of a health assessment tool. Authorization for the translation and validation was obtained from the author of the original scale (Benjamin Gierk). Data were collected at the dermatology outpatient clinic of the School of Medicine of *Universidade Estadual Paulista* (UNESP) in the city of Botucatu, state of São Paulo, Brazil, between March 2017 and May 2019.

The eligibility criteria were a diagnosis of psoriasis (any subtype and any degree of severity), age 18 years or older, undergoing treatment at the UNESP dermatology outpatient clinic and written consent to participate in the study. Individuals unable to complete the questionnaires used in the study were excluded.

Data collection involved the use of a questionnaire addressing sociodemographic characteristics, the Skindex-16 measure for the assessment of quality of life related to skin disorders and the Somatic Symptom Scale translated into Brazilian Portuguese (SSS-8-BRA).

Skindex-16 is a multidimensional instrument with three dimensions: symptoms (Items 1 to 4), emotions (Items 5 to 11) and functioning (Items 12 to 16). Each item is scored on a seven-point scale ranging from 0 (never) to 6 (always) based on the frequency with which the respondent was bothered due to her/his skin condition in the previous seven days. All answers are transformed into a linear scale ranging from 0 to 100 points. The score of each of the dimensions is calculated, with higher scores denoting poorer quality of life [3].

The SSS-8 is a short, eight-item version of the Patient Health Questionnaire (PHQ-15) [6]. The score ranges from 0 to 32, depending on the burden of symptoms, and is classified as follows: 16–32 points = very high burden; 12 to 15 points = high burden; 8 to 11 points = medium burden; 4 to 7 points = low burden; and 0 to 3 points = minimal burden [6].

### Translation and cultural adaptation procedures

The SSS-8-BRA was translated following international guidelines. The translation and validation process comprised six stages: 1 – initial translation by two independent translators; 2 – synthesis of translations; 3 – back-translation into original language; 4 – analysis of expert committee; 5 – pretest; and 6 – validation [15].

**Fig. 1:**
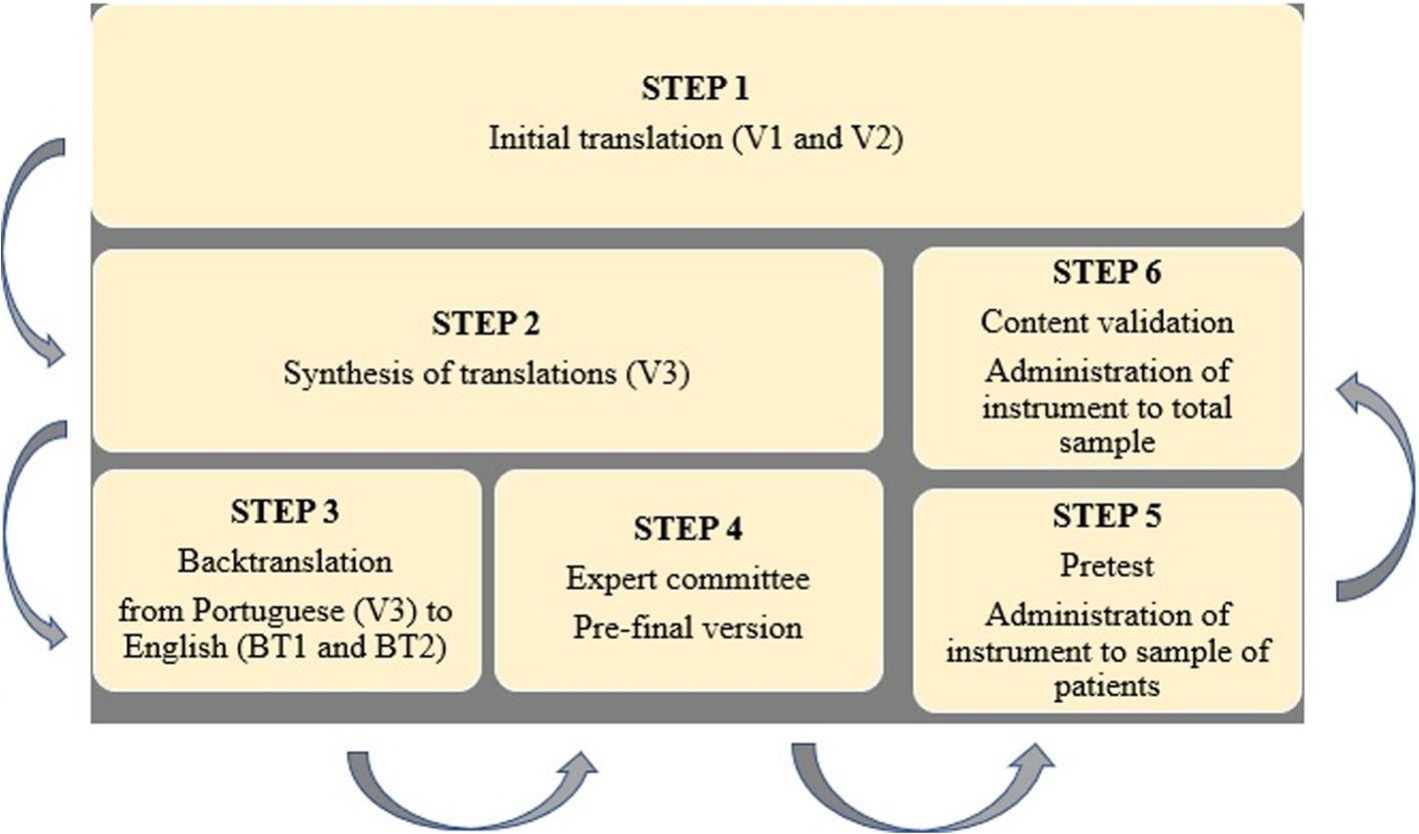
Flowchart of stages of cross-cultural adaptation and content validation of SSS-8-BRA for use in Brazil

The first step was performed by two translators whose native language was that to which the instrument was translated and who were fluent in the language of the original instrument. The second step was performed by the main researchers of the present study to finalize the new version of the instrument in the language proposed for the study. The third step was the back-translation by translators fluent in the language to which the instrument was translated and the language of the original version of the scale. In this step, the translators had no previous knowledge of the original instruments to avoid any influence on the backtranslation process. Next, the expert committee analyzed all versions with regard to semantic and conceptual equivalence (Step 4) for the establishment of the pre-final version to be administered to a sample of patients. In Step 5, patients were invited to participate in the pretest and asked to make suggestions to improve the understanding of the scale. Content validation was performed in Step 6. The steps of the process are displayed in Fig. 1.

#### Content validity

Content validation was performed by the expert committee composed of five physicians – all with previous experience in the validation of health measures – who evaluated the items with regards to clarity, relevance, pertinence and representativity.

#### Construct validity

Confirmatory factor analysis was performed to assure the one-dimensionality of the scale. The adequacy was assessed using the root mean square error of approximation (RMSEA), comparative fit index (CFI), and Tucker-Lewis index (TLI): RMSEA < 0.08, CFI > 0.9, and TLI > 0.95. The minimum value of discrepancy function based on Chi-squared (CMIN) and CMIN/df were also estimated. In addition, the standardized loading of the itens should be greater than 0.4. The parameters were assessed through a DWLS estimator using a robust error calculation [16]. Next, the convergent validity test was performed, in which the correlation between the Skindex-16 and SSS-8-BRA was determined using Spearman’s correlation coefficient, which was interpreted as follows: < 0.4 = weak correlation; 0.4 to 0.6 = moderate correlation; and > 0.6 = strong correlation [17].

Based on classic theory, a sample of up to 20 individuals is desirable for each item on an instrument to ensure adequate exploratory factor analyses in questionnaire validation tests [18]. As the SSS-8 is composed of eight items, the suggested sample dimensioning encompasses a range between 80 and 160 individuals. However, the scale was administered to 300 individuals.

#### Internal consistency

Cronbach’s alpha was used for the evaluation of the internal consistency of the instrument, for which coefficients higher than 0.70 were considered acceptable [19].

The data were tabulated on MS Excel 2010 spreadsheets and analyzed using the SPSS 25v and Factor 10.10 software programs. The level of significance was set at 5% (*p* < 0.05).

This study received approval from the institutional review board of *Universidade Estadual Paulista Júlio de Mesquita Filho* (certificate number: 1.908.794). This study was conducted in accordance with the Declaration of Helsinki. All of the patients were informed of the benefits and risks related to the study and provided their written informed consent for the study.

The methodological quality of study will be assessed using the COnsensus-based Standards for the selection of health Measurement Instruments (COSMIN) [20].

## Results

### Translation and back-translation of SSS-8

The translation of the original version of the scale into Brazilian Portuguese was performed by two bilingual (Portuguese and English) Brazilian translators, neither of whom had previous knowledge of the instrument. The translated versions were denominated V1 and V2 and were analyzed by the researchers, who then created a single version synthesized from the two translations denominated V3. This synthesized version was then back-translated into English by two English language teachers fluent in both languages (English and Portuguese). The back-translated versions were denominated BT1 and BT 2.

### Content validation and pretest

For content validation, the expert committee was comprised of five physicians – all with previous experience in the validation of health measures –, who analyzed the items with regards to relevance, pertinence and representativity. All judges agreed that the items met these criteria and no suggestions for changes were made.

For the pretest and semantic analysis, the scale was administered to 30 patients with psoriasis who were undergoing treatment at the dermatology outpatient clinic of the university where this study was developed. There were no refusals to participate.

### Psychometric validation

In the psychometric validation step, a convenience sample was composed of 300 outpatients. Women (*n* = 158; 53%), married/cohabitating individuals (*n* = 218; 73%), individuals with a primary school education (*n* = 162; 54%) and those with a family income between R$ 1100 and R$ 3000 (*n* = 173; 58%) predominated in the sample. These data are displayed in Table 1.

**Table 1:** Clinical and demographic characteristics of individuals with psoriasis (*n* = 300)

Variables	Values ***(n/%)***
n – total	300
Sex	
Female	158 (53)
Male	142 (47)
Age – mean (*SD*) in years	51.9 (13.8)
BMI – mean (*SD*) – kg/m^2^	28.8 (5.7)
Thin/undernourished (< 18.5 kg/m^2^)	2 (1)
Ideal range (18.5–24.9 kg/m^2^)	69 (23)
Overweight (25.0–29.9 kg/m^2^)	114 (38)
Obesity (≥30 kg/m^2^)	115 (38)
Family income – *n (%)*	
Up to R$ 1000	43 (14)
R$ 1100 to R$ 3000	173 (58)
R$ 3100 to R$ 5000	51 (17)
More than R$ 5000	33 (11)
Schooling – *n (%)*	
Primary school	162 (54)
High school	88 (29)
Higher education	50 (17)
Marital status – *n (%)*	
Single/Widowed	82 (27)
Married/Cohabitating	218 (73)
Age at onset of disease – mean (SD) in years	35.6 (14.1)
Disease duration – median (25th–75th p) in years	14 (7–24)
Joint psoriasis – *n* (%)	35 (12)
Nail psoriasis – *n (%)*	61 (20)

*SD* standard deviation, *BMI* body mass index, *p* percentile

### Clinical validity

**Fig. 2:**
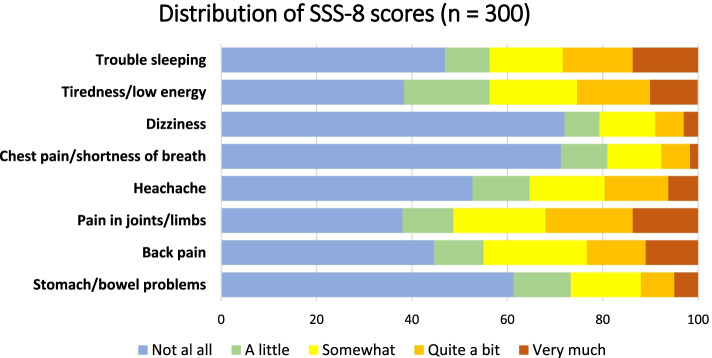
Distribution of SSS-8 scores

The distribution of the scores of the SSS-8 items was asymmetrical for the majority of items (Fig. 2). Moreover, more than 60% of the answers for the items “pain in stomach/gut”, “chest pain/shortness of breath” and “dizziness” were “not at all”, indicating a floor effect.

### Construct validation

**Fig. 3:**
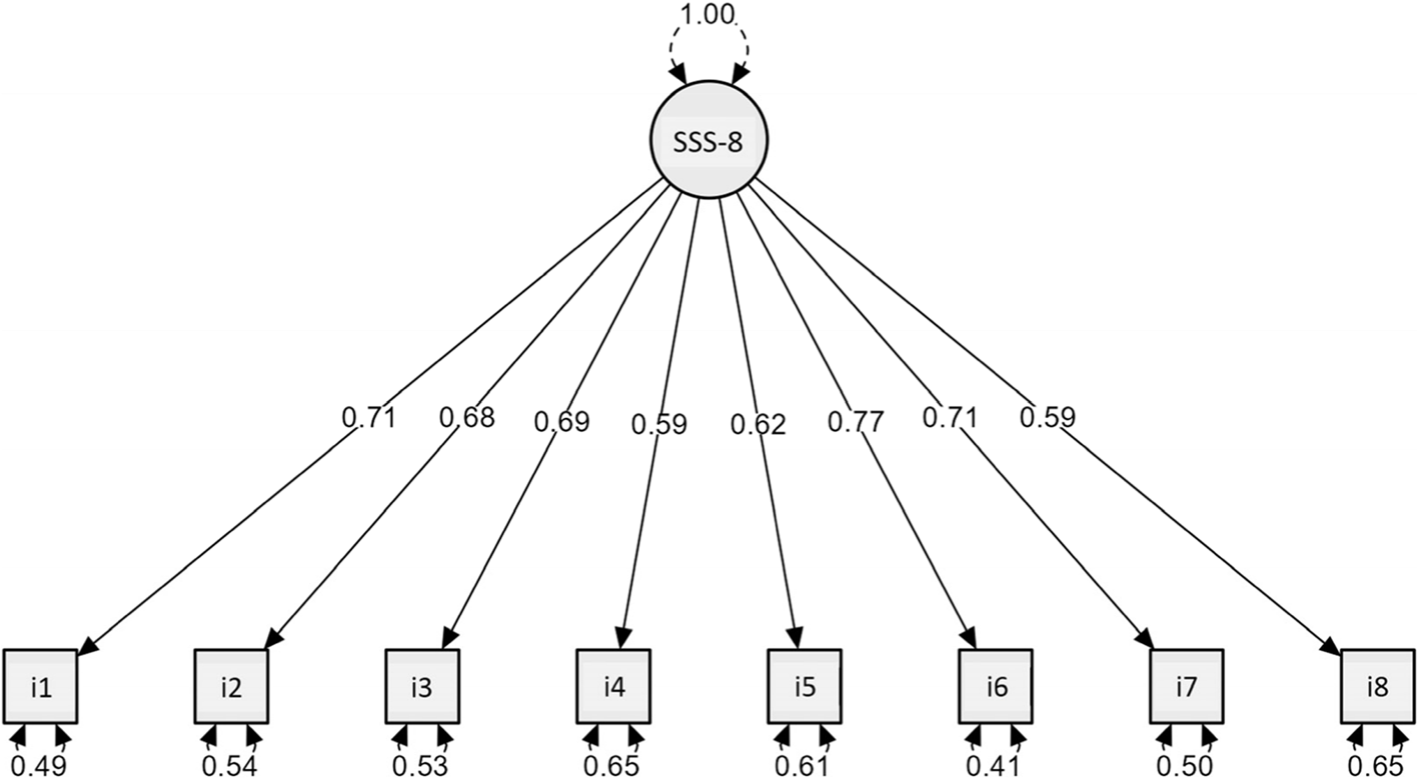
Confirmatory factor analysis path diagram and standardized loading of the itens

The one-dimensionality of the instrument was indicated by the confirmatory factor analysis. The fit indexes results are: RMSEA (0.015; *p* = 0.932), CFI (0.999), TLI (0.999). CMIN was 21.25 (*p* = 0.038), and CMIN/df was 1.06. The standardized loadings of the items resulted ≥0.58 (Fig. 3). The latent variable explained by the unidimensional factor was 59%.

### Convergent validity

In the absence of an equivalent instrument to serve as the “gold standard” that met standards of excellence for the study, the Skindex-16 quality of life scores of the patients (symptoms dimension) were used for the analysis of convergent validity. Spearman’s correlation coefficients calculated for the SSS-8-BRA and the dimensions of Skindex-16 (Symptoms, Emotions, Functioning) resulted: 0.46, 0,38, and 0.36 (*p* < 0.01).

### Internal consistency

In the analysis of internal consistency, Cronbach’s alpha for the SSS-8-BRA was 0.81 (95% CI: 0.77 to 0.84).

## Discussion

The present study involved the translation, cultural adaption and determination of the psychometric properties of the SSS-8-BRA, which can facilitate the exchange of experiences and the broad dissemination of knowledge among scientific communities [21].

The validation process is composed of steps that establish whether an assessment tool measures what it intends to measure. In this process, convincing evidence is expected, demonstrating that the instrument is useful [22].

In the present study, although the content validation was performed by individuals with high education levels, the schooling of the patients did not affect their understanding of the instrument, underscoring the importance of cross-cultural adaptation in the translation of health assessment tools.

The SSS-8 is a measure of the burden of somatic symptoms. The original study conducted in Germany involved 2510 participants from the general population aged 13 years or older [6]. Confirmatory factor analysis was performed in the original study, which tested two models theoretically derived from previous studies [6]. In contrast, exploratory factor analysis was employed in the present investigation, the aim of which was to understand how the items were grouped.

This study involved 300 participants. The sample size for confirmatory factor analysis is a matter of discussion. By the way as long the model fits better, the sample size can be restricted as well. Thus, according to given RMSEA value (0.015 vs at least 0.8), the minimum sample size can be estimated as 221 participants, for a power of 90% [23]. Here, the sample size was 37.5 times the number of variables for SSS-8, which surpasses all the recommendations for exploratory and one-factor confirmatory factor analysis [24].

In the original study, bivariate correlations were tested for the determination of construct validity of the SSS-8 using the PHQ-2, the Generalized Anxiety Disorder-2 scale, and a general health visual analog scale and significant correlations were found [6]. In the present study, construct validity was tested through convergent validity, with the investigation of the correlation between the SSS-8-BRA and the Skindex-16, for which a significant moderate correlation was found (Spearman’s coefficient: 0.458).

Internal consistency of the SSS-8-BRA was evaluated using Cronbach’s alpha, which is a highly relevant method for measuring the reliability of scales with various items [19]. Despite being the most widely used method for the evaluation of internal consistency, there is no consensus on its interpretation. While some studies report that values higher than 0.7 are ideal, some authors consider lower values (close to 0.60) to be acceptable [25]. One should bear in mind that Cronbach’s alpha coefficients are strongly influenced by the number of items on a measurement instrument [26]. A small number of items per domain on an instrument can diminish the alpha values, thereby affecting internal consistency [27]. In the present study, Cronbach’s alpha coefficient for the SSS-8-BRA was 0.81, which is the same as that found for the original version of the scale, demonstrating that reliability was maintained [6].

Some diseases have a single symptom. The SSS-8-BRA is not indicated in such cases, as this measure was created for multi-symptom diseases (chronic and systemic conditions with several symptoms at the same time). Therefore, the present study involved a representative sample with psoriasis, which is a systemic disease with several comorbidities.

The SSS-8 measures the burden of somatic symptoms as a subjective, patient-reported outcome without making any suppositions regarding the cause [6]. This scale is potentially useful in numerous medical situations, as the scores can serve as quantitative markers of the burden of somatic symptoms in patients with chronic conditions, such as coronary disease [28] or mental disorders, who often experience somatic symptoms that exert negative impacts on psychological and social functioning and, consequently, quality of life [29].

The limitation of the present study was the lack of the item “pruritus” in the composition of the SSS-8, which is an important symptom in dermatology and particularly in psoriasis. In addition, this study was performed in a single center, which restricts the generalization of its validation for other populations who live in an extensive country, as Brazil. However, the SSS-8-BRA is efficient, brief, quick to administer, reliable and valid and can be answered completely by the patient.

## Conclusion

The SSS-8 translated and validated for Portuguese is a simple, valid, reliable tool that is quick to administer for the assessment of somatic symptoms in the Brazilian population.

## Supplementary Information

**Additional file 1.** Somatic Symptom Scale-8 (SSS-8-BRA).

## Data Availability

The datasets used and/or analyzed during the current study are available from the corresponding author on reasonable request.
