# Supplementary material for: Translation, cultural adaptation and validation of the Somatic Symptom Scale-8 (SSS-8) for the Brazilian Portuguese language
Source: BMC Prim Care. 2022 Sep 5;23:222. doi: 10.1186/s12875-022-01836-2 (PMC9442982; doi:10.1186/s12875-022-01836-2)
Supplement: Supplementary file 1 — Additional file 1. Somatic Symptom Scale-8 (SSS-8-BRA). [file 12875_2022_1836_MOESM1_ESM.docx]

Somatic Symptom Scale-8 (SSS-8-BRA).

| In the past **7 days,** how often have you been bothered by any of the following symptoms? | | | | | |
| --- | --- | --- | --- | --- | --- |
|  | Not at all | A little | Somewhat | Quite a bit | Very much |
| Stomach or bowel problems | 0 | 1 | 2 | 3 | 4 |
| Back pain | 0 | 1 | 2 | 3 | 4 |
| Pain in your arms, legs or joints | 0 | 1 | 2 | 3 | 4 |
| Headache | 0 | 1 | 2 | 3 | 4 |
| Chest pain or shortness of breath | 0 | 1 | 2 | 3 | 4 |
| Dizziness | 0 | 1 | 2 | 3 | 4 |
| Tiredness or low energy | 0 | 1 | 2 | 3 | 4 |
| Trouble sleeping | 0 | 1 | 2 | 3 | 4 |

Copyright © 2014, American Medical Association. All rights reserved
